# Search for germline alterations in CDKN2A/ARF and CDK4 of 42 Jewish melanoma families with or without neural system tumours

**DOI:** 10.1038/sj.bjc.6602629

**Published:** 2005-05-31

**Authors:** C Marian, A Scope, K Laud, E Friedman, F Pavlotsky, E Yakobson, B Bressac-de Paillerets, E Azizi

**Affiliations:** 1 Service de Génétique, Institut Gustave Roussy, Villejuif, France; 2 Department of Dermatology, Sheba Medical Center, Tel-Aviv University, Israel; 3 The Susanne Levy Gertner Oncogenetics Unit, Sheba Medical Center, Tel-Aviv University, Israel; 4 Molecular Cell Biology Laboratory, Department of Internal Medicine C, Sheba Medical Center, Sackler Faculty of Medicine, Tel-Aviv University, Israel

**Keywords:** Ashkenazi Jewish melanoma families, neural system tumours, CDKN2A/ARF, CDK4, germline mutations, inherited predisposition

## Abstract

To gain insight into the molecular mechanisms involved in the inherited predisposition to melanoma and associated neural system tumours, 42 Jewish, mainly Ashkenazi, melanoma families with or without neural system tumours were genotyped for germline point mutations and genomic deletions at the CDKN2A/ARF and CDK4 loci. CDKN2A/ARF deletion detection was performed using D9S1748, an intragenic microsatellite marker. Allele dosage at the *p14*^*ARF*^ locus was analysed by quantitative real-time PCR employing a TaqMan probe that anneals specifically to exon 1*β* of the *p14*^*ARF*^ gene. For detecting point mutations, dHPLC and direct sequencing of the coding sequences of CDKN2A/ARF and CDK4 was used. No germline alterations in any of the tested genes were detected among the families under study. We conclude that in the majority of Ashkenazi Jewish families, the genes tested are unlikely to be implicated in the predisposition to melanoma and associated neural system tumours.

Familial cutaneous malignant melanoma is a genetically heterogeneous condition linked to chromosome 9p21 in many, but not all families (Hussussian *et al*, 1994; Greene, 1999). To date, germline mutations in two high penetrance genes have been identified in some of these families, *CDKN2A/ARF* and *CDK4*. The *CDKN2A/ARF* gene encodes two distinct proteins, p16^INK4^ and p14^ARF^, the result of alternative splicing of exons 1*α* and 1*β*, respectively. The p16^INK4^ protein, which belongs to the INK4 family of cyclin-dependent kinase inhibitors, plays a key role in arresting cell cycle progression at the G1 phase by inhibiting cyclins CDK4 and CDK6 and subsequently blocking their ability to phosphorylate the retinoblastoma protein Rb (Chin *et al*, 1998). The p14^ARF^ protein is also involved in cell cycle regulation by interacting with different substrates in the p53 pathway (Pomerantz *et al*, 1998), and by binding to MDM2, also in the Rb pathway with resultant cell cycle arrest in both G1 and G2 phases (Xiao *et al*, 1995; Weber *et al*, 1999; Momand *et al*, 2000). In all, 20% of melanoma families were found to harbour genetic alterations at the *CDKN2A/ARF* gene (Goldstein, 2004). The other gene involved in familial predisposition to melanoma, *CDK4* is a proto-oncogene that promotes cell cycle progression by phosphorylating the Rb protein. Germline mutations in CDK4 were detected in three melanoma families (Zuo *et al*, 1996; Soufir *et al*, 1998).

The familial clustering of both melanoma and neural system tumours (NST) was first reported in 1993 by Kaufman *et al* (1993) in a single family with eight family members over three generations who were diagnosed with cutaneous melanoma, cerebral astrocytoma or both. Azizi *et al* (1995) surveyed 904 melanoma Jewish-Israeli patients for the occurrence of NST in their family pedigrees. Melanoma-affected members within families, as well as first and second-degree relatives, were found to be at an increased risk for developing NST. A total of 15 families with a clustering of melanoma and a variety of NST were identified, and 10 patients with two primary tumours, melanoma and NST, primarily meningioma were described (Azizi *et al*, 1995). Similar familial clustering of melanoma and NST was described in French (Bahuau *et al*, 1997) and Finnish families (Paunu *et al*, 2002). Recently, melanoma and NST association was confirmed by epidemiological and population-based studies in Scandinavia (O'Neill *et al*, 2002; Hemminki *et al*, 2003; Nielsen *et al*, 2004). The familial clustering of melanoma and NST has been recognised and designated as the Melanoma and Neural System Tumour syndrome (MM-NST) (OMIM # 155755), and in a small subset of melanoma-NST kindreds germline mutations, mainly deletions affecting the CDKN2A/ARF gene and cosegregating with both tumours, were described.

In the present study, 42 Jewish, mainly Ashkenazi, melanoma families with (*n*=24) or without NST (*n*=18) were genotyped for germline sequence alterations in the CDKN2A/ARF and CDK4 genes. Mutational screening of 24 families with co-occurrence of melanoma and NST is the largest analysis reported thus far.

## MATERIALS AND METHODS

### Patients

Jewish families with a history of melanoma and NST were recruited to the study. The inclusion criteria (based on OMIM's definition) were a minimum of two cancers in the pedigree, one being melanoma and the other NST, or an individual harbouring both tumours.

Additional 18 Jewish melanoma families without NST, having at least two or more individuals with melanoma, or multiple melanomas in a single family member – as minimal inclusion criteria – were also included.

The families had been recruited between the years 1997 and 2003. The study had been approved by the Institutional Ethics Committee of the Sheba Medical Center, Israel. All participants signed a written informed consent prior to being enrolled in the study. Demographic details, including country of birth of the probands, their parents and grandparents, were collected using a self-response questionnaire. Classification to ethnic groups was done according to the country of birth of the grandparents on both the maternal and paternal sides, provided that one or both parents were either from the same origin, or Israeli-born. Families with both sets of grandparents from Eastern and Central European countries were classified as Ashkenazi. Families originating from Spain, North-Africa, Balkans, or Iraq, Iran, Yemen and Egypt were classified as Sephardic. Every effort has been made to confirm the correct cancer type for affected members based on pathology report, patients' medical charts, operation reports and death certificate. When these were not available, information regarding tumours that was obtained by history from several family members but not confirmed by a pathology report was designated as histological type not specified. A dermatological examination assessing skin phenotype, atypical mole syndrome (AMS) score (Newton Bishop *et al*, 1994) and signs of dermato-heliosis was performed by one of three participating dermatologists and 10 ml of venous blood samples were withdrawn for DNA extraction.

### Genetic alterations detection

#### DNA preparation

Genomic DNA was extracted from peripheral blood leucocytes using the Puregene® Genomic DNA Isolation Kit (Gentra Systems, Minneapolis MN, USA), using the manufacturer's recommended protocol.

#### Mutation analysis

For detecting *CDKN2A/ARF* and *CDK4* gene coding region sequence alterations, exons 1*α*, 1*β* and 2 of *CDKN2A/ARF* and exon 2 of *CDK4* were screened by dHPLC (denaturing high performance liquid chromatography), by using PCR and dHPLC analysis conditions previously described (Laud *et al*, 2003). Briefly, PCR was carried out in a final volume of 20 *μ*l containing 100 ng genomic DNA, 1 × HotStar *Taq* DNA Polymerase buffer with 1.5 mM MgCl_2_ (Qiagen), 4 pmoles of each primer, 1 UI HotStar *Taq* DNA Polymerase (Quiagen) and 2.5 mM dNTPs. For PCR amplification of each exon, a touch down protocol was used as follows: initial denaturation and HotStar *Taq* Polymerase activation at 95°C for 15 min; six cycles of 30 s at 95°C, 30 s at 66°C (the annealing temperature decreasing by 2°C at every two cycles), 30 s at 72°C; followed by 40 cycles of 30 s at 95°C, 30 s at 60°C and 30 s at 72°C. Heteroduplex analyses were carried out on an automated dHPLC instrument (WAVE, Transgenomic, CA, USA). DNA samples with known germline mutations at *CDKN2A/ARF* locus were used as positive controls.

Samples displaying abnormal profiles were subsequently bidirectionally sequenced using the BigDye™ Terminator sequencing kit (Applied Biosystems, Foster City, CA, USA) according to the manufacturer's instructions on an ABI Prism 377 instrument (Applied Biosystems, Foster City, CA, USA).

#### CDKN2A/ARF deletion detection

Since CDKN2A/ARF deletions were previously identified in melanoma-NST families (Bahuau *et al*, 1998, Randerson-Moor *et al*, 2001), deletions were sought only in this subset of families (*n*=24). Deletion genotyping was performed using the D9S1748 microsatellite marker located adjacent to CDKN2A exon 1*β*. The PCR amplifications were carried out in a final volume of 25 *μ*l, the reaction mix containing: 1 × HotStar *Taq* DNA polymerase buffer with 1.5 mM MgCl_2_ (Qiagen, Chatsworth, CA, USA), 1 UI HotStar *Taq* DNA polymerase (Qiagen), 4 pmoles of each primer and 0.2 mM dNTPs. Primer sequences are available through The Genome Database (http://www.gdb.org). The forward primer was fluorescently labeled with the 6-FAM at its 5′ extremity. The PCR products were loaded on a 6%/7 M urea denaturing polyacrylamide gel in an ABI Prism 377 (Applied Biosystems, Foster City, CA, USA) device along with the ROX 350 (Applied Biosystems, Foster City, CA, USA) internal marker standard. Genotypes were analysed using the GeneScan software (Applied Biosystems, Foster City, CA, USA). Since the homozygous status could possibly be due to the loss of an allele, homozygous samples were further analysed for allele dosage (Barrois *et al*, 2004) at the p14ARF locus by quantitative real-time PCR using an ABI Prism 7700 instrument (Applied Biosystems, Foster City, CA, USA). A TaqMan probe that anneals specifically to the exon 1*β* of the p14ARF gene, marked with a fluorescent reporter dye (FAM) and a quencher dye (TAMRA), was used. By calculating the ratio initial copy number of p14ARF/initial copy number of GAPDH, we obtained the normalized gene dose. The PCR was performed in triplicate for each sample in a final volume of 50 *μ*l, the reaction mix containing for the GAPDH gene 1 × TaqMan Universal Master Mix (Applied Biosystems, Foster City, CA, USA), 15 pmoles of each primer and probe and 25 ng DNA. For the exon 1*β* of CDKN2A, same quantities were used, with the exception of the TaqMan Universal Master Mix which was replaced by 1 × TaqMan PCR Core Reagent Buffer (Applied Biosystems, Foster City, CA, USA), 2.5 mM dNTPs, 5% glycerol, 5 mM MgCl2 and 1.25 UI AmpliTaqGold DNA polymerase (Applied Biosystems, Foster City, CA, USA). Amplification conditions were: 2 min at 50°C, 10 min at 95°C (20 min for p14ARF) followed by 40 cycles of 15 s at 95°C and 1 min at 60°C. We used as positive control the haploid cell line HL60 kindly provided by Juliette Moor and Julia Newton Bishop from Genetic Epidemiology Division, Cancer Research UK, St James's University Hospital, Leeds, UK (Randerson-Moor *et al*, 2001).

## RESULTS

### Clinical features of the study participants

The study population included (a) 25 probands and 11 unaffected relatives from 24 families with pedigrees displaying cutaneous melanoma and NST and (b) 20 probands from 18 melanoma families without NST, among them 13 families with pedigrees containing two or more melanoma-affected individuals and five families containing individuals with multiple melanomas. Notably, the clinical features of two families (#107 and #121) have already been described earlier (Azizi *et al*, 1995). Distribution by tumour type, number of tumours and family affiliation is presented in Tables 1 and 2 (melanoma-NST families) and 3 (familial melanoma).

**Figure 1:**
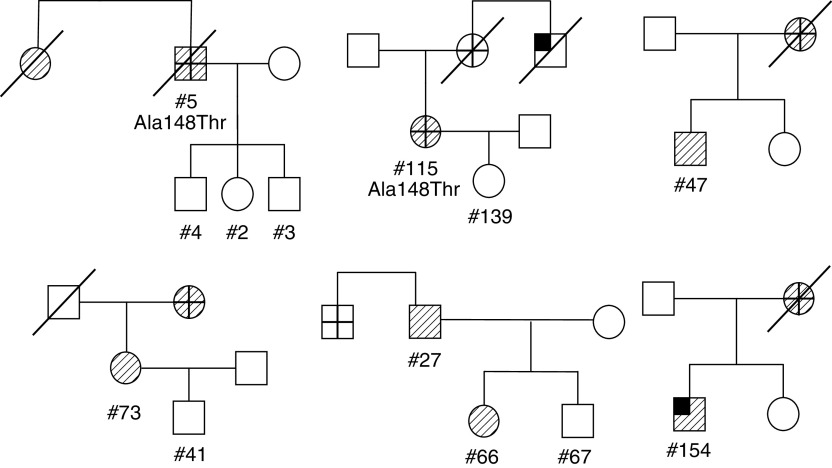
Representative pedigrees of six Jewish families with melanoma and neural system tumours (NST). Family codes of presented pedigrees (from left-to-right, top-to-bottom) – #110, #116, #118, #114, #109, #101. Striped squares and circles indicate male and female subjects with melanoma, respectively. Crossed symbols indicate individuals with neural system tumours and upper quarter filled symbols represent the presence of other tumours. Sample DNA code number and sequence alterations are indicated.

Among the melanoma-NST pedigrees, in 10/24 (42%) families there were two melanoma or two NST cases, and in the others in this category, there were one melanoma and one NST in each family (Table 1). The male : female (M : F) ratio among the affected cases was 1. The melanoma and NST were diagnosed at the age range of 22–74 years and 10–86 years, respectively. Of the 24 families in this subgroup, 22 were of Ashkenazi origin, one out of 24 was Sephardic (#107), and one out of 24 heterogeneous (#121). Major phenotypic features of the patients, available in 13 out of 25 probands, were variable with no specific pattern. Additional cancers that were reported in this series included colon cancers in four families; breast cancer in three families; lung cancer in two families; liver cancer in two families; and renal, gastric, laryngeal, pleural and nonmelanoma skin cancer each in one family (Table 2). Examples of pedigrees showing melanoma families with NST are presented in Figure 1.

**Figure 2:**
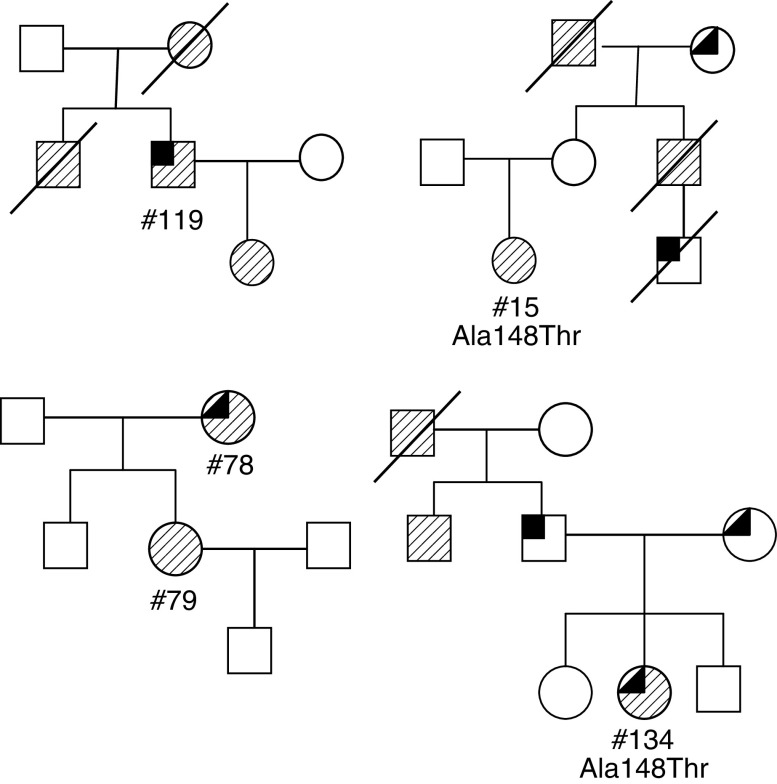
Representative pedigrees of four Jewish melanoma families without neural system tumours. Family codes of presented pedigrees (from left-to-right, top-to-bottom) – #312, #321, #325, #335. Striped squares and circles indicate male and female subjects with melanoma, respectively. Upper quarter filled symbols represent individuals with other tumours. Sample DNA code number and sequence alterations are indicated.

Among the 18 melanoma families without NST (Table 3), the M : F ratio among the melanoma patients was 1 : 2, and the age at diagnosis was in the range of 25–88 years. Of 18 families in this series, 17 were of Ashkenazi origin. Family #321 was of heterogeneous Romanian (Ashkenazi)/Turkish-Greek (Sephardic) origin. Major phenotypic features of the melanoma patients, not available for two out of 20 probands included dermato-heliosis and solar keratosis (15 out of 18), freckles (15 out of 18) and AMS ⩾2 (seven out of 18). Additional cancers that were reported in this series included nonmelanoma skin cancer in three families; prostate cancer in three; breast cancer in five; pancreatic cancer in one; transitional cell carcinoma in one and lymphoma in one. Examples of pedigrees showing melanoma families without NST are presented in Figure 2.

### Mutational analyses of the CDKN2/ARF and the CDK4 genes for point mutations

Overall, nine samples displayed different chromatographic profiles. Sequence analyses revealed a G to A transition at position 442 leading to a missense mutation at codon 148 (Ala148Thr) in all nine patients: patients #5, #83 and #115, all unrelated, among the melanoma-NST families (Table 4); and patients #15, #111, #114, #116, #124, #134, all unrelated among the melanoma families without NST (Table 5).

### Detection of CDKN2A/ARF gene deletions

A total of 30 individuals among the melanoma-NST families were genotyped using the D9S1748 microsatellite marker, located adjacent to exon 1*β* of *CDKN2A/ARF* gene on chromosome 9p21. A total of 12 samples displayed a heterozygous status, that is, two alleles without genomic deletion (Table 4); And 18 samples displaying homozygous profiles for this locus were selected for further analysis, since the homozygous status could indicate the loss of an allele by a large deletion encompassing exon 1*β*. Gene dosage for these samples, as well as six samples not analysed for the D9S1748 microsatellite marker, showed no deletions, therefore, all individuals presented two alleles (Table 4).

## DISCUSSION

In the present study, no *bona fide* pathogenic germline alterations were identified in the CDKN2A/ARF and CDK4 loci among 42 Jewish, primarily Ashkenazi Israeli families, with a seemingly inherited predisposition to cutaneous melanoma, and in some, clustering of melanoma with NST, for deletions and point mutations in the CDKN2A/ARF and CDK4 loci. The only sequence variation identified in nine DNA samples was G to A transition at position 442 leading to a missense mutation at codon 148 (Ala148Thr). The Ala148Thr missense mutation is considered as a polymorphism based on several observations: it has been previously reported in individuals from the general, average risk, population in ethnically diverse groups: 8% of the Jewish population (Yakobson *et al*, 2000), 4% of the population in Utah (Kamb *et al*, 1994) and 5% in the UK population (Bertram *et al*, 2002). Furthermore, this missense mutation did not segregate with the phenotype in familial melanoma (Hussussian *et al*, 1994; Harland *et al*, 1997), and is situated outside the critical four ankyrin repeat domains of p16, and thus does not appear to have any effect *in vitro* on binding to CDK4 (Ranade *et al*, 1995; Lilischkis *et al*, 1996; Harland *et al*, 1997). Ala148Thr was further analysed in twin studies as a candidate low penetrance polymorphism enhancing the risk of melanoma by increasing AMS score (Zhu *et al*, 1999). However, the rate of this polymorphism in families with atypical mole phenotype was similar to general population (Bertram *et al*, 2002). Thus, Ala148Thr is considered a p16 polymorphism and not a pathogenic mutation.

Genetic mutations at the CDKN2A gene have been identified in 20% of melanoma families, most of these in exons 1*α* and 2 (Goldstein, 2004). Several studies also implicated the ARF gene as underlying melanoma predisposition. A 16 bp insertion in exon 1*β*, which affects the function of p14 but not p16, was described in a patient with multiple primary melanomas (Rizos *et al*, 2001). A splice site mutation in exon 1*β*, which results in p14 haploinsufficiency, was also reported in two affected persons from melanoma kindred (Hewitt *et al*, 2002). The negative mutation detection results in the present study among Jewish Ashkenazi melanoma families are not in line with the expected mutation rate, based on previously reported data in non-Jewish populations.

Among families with melanoma-NST association, the loss of function of CDKN2A/ARF can be a predisposing factor. Segregation analysis of two melanoma-NST French families showed hemizygous germline deletion that ablated CDKN2A/ARF gene (Bahuau *et al*, 1998). Analysis of 11 families with two or more cases of glioma revealed a hemizygous germline deletion in CDKN2A in one family with both glioma and melanoma (Tachibana *et al*, 2000). In another melanoma family with NST (mainly astrocytoma), deletion was found in the CDKN2A/ARF exon 1*β*. The deletion, leading to loss of ARF function, did not affect the coding region of p16 protein (Randerson-Moor *et al*, 2001). Finally, a splice site substitution mutation trimming CDKN2A exon 2 and severely affecting both p16INK4A and p14ARF was described in a family with melanomas, neurofibromas and multiple dysplastic nevi (Petronzelli *et al*, 2001). Constitutional CDKN2A locus alterations, somatic point mutations and deletions at CDKN2A were identified in NST (Ueki *et al*, 1996; Bostrom *et al*, 2001; Ghimenti *et al*, 2003). Evidence was presented that deletion in ARF may be the underlying cause in the development of melanoma and NST (Randerson-Moor *et al*, 2001).

Yet, the lack of mutations at the genes analysed in this series of melanoma families with NST is commensurate with data from other melanoma-NST families genotyped for mutations: in a family with melanoma and optic nerve glioma, no mutations were identified in the *CDKN2A* gene (Alao *et al*, 2002). Analysis of Swedish patients with multiple primary melanomas and NST was negative for the *CDKN2A* founder mutation *113insArg*, which usually explains all CDKN2A-associated familial melanoma in Sweden (Nielsen *et al*, 2004).

It is unlikely that the familial clustering of melanoma and NST is due to chance, as both tumours are relatively rare cancers in Israel, with age standardised incidence rates of 7.7 and 8.4 per 10^5^, respectively, in female subjects, and 7.6 and 10.3 per 10^5^, respectively, in male subjects (Azizi *et al*, 1995). All the melanoma families with NST in the present series withstand the OMIM criteria for the melanoma-NST syndrome. Yet, without identifying a mutation, cosegregating within the current series of families with both melanoma and NST, we cannot unequivocally determine the proportion of families that truly represent melanoma-NST syndrome. However, 10 families (42% of the series), having at least two melanoma or two NST probands, are strongly suggestive of an inherited predisposition for developing melanoma and NST.

Lack of germline mutations in the CDKN2A/ARF and CDK4 loci has been recently reported by Loo *et al* (2005) among 22 Ashkenazi Jewish families with an apparent inherited predisposition to melanoma. Taken together with the data reported herein, it appears that in over 60 Ashkenazi Jewish melanoma families, no germline alteration in CDKN2A/ARF and CDK4 loci underlie the apparent predisposition. One caveat to the present study that should be pointed out is that germline alterations in noncoding regions such as intronic and promoter sequences not screened in the present and in previous studies cannot be ruled out as contributing to familial melanoma.

In conclusion, in the majority of Ashkenazi Jewish families with an inherited predisposition to melanoma with or without NST, CDKN2A/ARF and CDK4 loci are unlikely to be implicated in the predisposition to melanoma and the associated neural system tumours.

## Figures and Tables

**Table 1:** The distribution of melanoma-NST pedigrees according to number of tumours

**No. of tumours^a^**	**Family #**	**Total *n* (%)**
NST × 2 and MM × 2	120	1 (0.5)
		
NST × 2 and MM × 1	116, 122, 113	3 (12.5)
		
MM × 2 and NST × 1	105, 109, 110 114, 118, 101	6 (25.0)
		
MM × 1 and NST × 1	112, 117, 119 102, 103, 104 106, 108, 111 115, 123, 124 107, 121	14 (58.0)
		
	Total	24 (100.0)

a MM=melanoma, NST=neural system tumours.

**Table 2:** Distribution of melanoma-NST pedigrees by tumour type and family affiliation

	**Proband**					**Affected relatives**			**Unaffected relatives**		
**Family #**	**DNA#**		**MM (a)**	**NST (b)**	**Additional cancers**	**MM**	**NST (b)**	**Additional cancers**			
**(*n*=24)**	**(*n*=25)**	**Gender**	**(Age at diagnosis)**			**(Age at diagnosis)**			**DNA#**	**Gender**	**Age**
116	115	F	68	64 (c)	BCC (66)	—	Mother	Lung (uncle)	139	F	35
					SCC (69)		(47) (x)				
120	218	M	51	51 (c)	BCC	—	—	Pleura (father)	—	—	—
	219	M	—	16 (b)	brother						
122	221	M	51	51 (g)	—	—	Father (84) (x)	Colon (mother)	—	—	—
110	5	M	52	45 (f)	—	Sister (50)	—	—	4	M	15
									2	F	20
									3	M	21
105	88	F	58	68 (c)	—	—	—	—	—	—	—
			67								
113	165	M	57	19 (x)	BCC	—	—	Lung (mother)	194	M	65
				25				Liver (cousin)			
112	169	F	63	63 (e)	—	—	—	—	—	—	—
117	153	F	55	56 (c)	—	—	—	Breast (mother)	—	—	—
109	27	M	67		—	—	Brother (a)	—	67	M	45
	66	F	40		Daughter						
114	73	F	22		—	Mother (48)	Mother (51,61) (e)	—	41	M	14
118	47	M	23		—	Mother (43)	Mother (51) (b)	—	—	—	—
101	154	M	32		BCC (37)	Mother (65)	Mother (68) (f)	—	185	F	45
119	—	—	—		—	Aunt	Sister (d)	Breast (sister, mother)	12	M	53
102	109	M	67		—	—	Grandson (24) (e)	—	133	F	45
103	6	M	40		—	—	Mother (71) (a)	Colon (mother) BCC/SCC Larynx (father) Liver (grand-mother)	—	—	—
104	13	F	67		Colon	—	Brother (a)	Colon (brother)	—	—	—
106	160	M	74		—	—	Father (68) (x)	—	166	M	75
108	131	M	67		—	—	Brother (57) (x)	—	—	—	—
111	113	F	66		—	—	Mother (x)	—	—	—	—
115	137	F	24		—	—	Grand-mother (86) (x)	—	—	—	—
123	222	F	56		—	—	Father (84) (x)	Colon (mother)	—	—	—
124	223	M	36		—	—	Cousin (30) (x)	Renal (father) Gastric (aunt)	—	—	—
107	83	F	43		—	—	Daughter (10) (h)	—	—	—	—
121	220	F	37		—	—	Grand-father (x)	Breast (mother)	—	—	—

a MM=melanoma.

b NST=neural system tumours, (a)=glioblastoma multiforme, (b)=oligodendroglioma, (c)=meningioma, (d)=glioma, (e)=neurilemmoma, (f)=malignant peripheral schwannoma, (g)=brain germinoma, (h)=medulloblastoma, (x)=NST, pathologic type unspecified.

**Table 3:** Distribution of Pedigrees of melanoma families without neural system tumours, by tumour type and family affiliation

	**Patient**				**Affected relatives**	
**Family #** **(*n*=18)**	**DNA# (*n*=20)**	**Gender**	**MM (a)**	**Additional cancers**	**MM**	**Additional cancers**
			**(Age at diagnosis)**		**(Age at diagnosis)**	
302	104	F	74	—	Cousin (72)	—
303	103	M	49 (7 primary MM)	BCC SCC	Brother	—
305	100	F	74	BCC (75)	Nephew	—
309	112	F	52	—	—	Pancreas (father)
310	111	F	45 64	—	—	—
311	105	F	48	—	Sister	Breast (mother)
312	119	M	68	Prostate (68)	Mother (83) Brother (65) Daughter (19)	—
317	116	F	60	—	Grandmother	—
319	114	M	36	—	Cousin	—
321	15	F	25	—	Grandfather (61) Uncle	TCC (cousin) Breast (grandmother)
322	124	M	66	Prostate	—	Breast (mother)
			67	(67)		
325	78	F	61 61	Breast (56)	—	—
	79	F	33	daughter		
327	121	M	86	—	—	—
	135	F	88	sister		
329	122	F	47 62	—	—	Lymphoma (mother)
330	140	F	67 71	—	Daughter	—
335	134	F	25	Breast	Uncle Grandfather	Breast (mother) BCC (father)
337	141	M	64 65	Prostate (56)	—	—
338	138	F	52 53	BCC (47)	Daughter (32)	—

a MM=melanoma.

**Table 4:** Mutation detection analysis in p16, p14 and CDK4 genes of melanoma-NST pedigrees (*n*=24)

**Family # (*n*=24)**	**DNA # (*n*=36)**	**P16 sequencing analysis**	**P14 deletion analysis by D9S1748**	**Quantative TaqMan analysis of p14**	**p14 sequencing**	**CDK4 sequencing**
101	154	WT	Hmz	2n	WT	WT
	185^a^	WT	Htz	ND	WT	WT
102	109	WT	Htz	ND	WT	WT
	133^a^	WT	Hmz	2n	WT	WT
103	6	WT	Hmz	2n	WT	WT
104	13	WT	Htz	ND	WT	WT
105	88	WT	Hmz	2n	WT	WT
106	160	WT	Htz	ND	WT	WT
	166^a^	WT	Htz	ND	WT	WT
107	83	Ala148Thr	Hmz	2n	WT	WT
108	131	WT	Htz	ND	WT	WT
109	27	WT	Htz	ND	WT	WT
	66	WT	Hmz	2n	WT	WT
	67^a^	WT	Hmz	2n	WT	WT
110	5	Ala148Thr	Hmz	2n	WT	WT
	4^a^	WT	Hmz	2n	WT	WT
	2^a^	WT	Hmz	2n	WT	WT
	3^a^	WT	Htz	ND	WT	WT
111	113	WT	Hmz	2n	WT	WT
112	169	WT	Htz	ND	WT	WT
113	165	WT	Hmz	2n	WT	WT
	194^a^	WT	Hmz	2n	WT	WT
114	73	WT	Hmz	2n	WT	WT
	41^a^	WT	Htz	ND	WT	WT
115	137	WT	Hmz	2n	WT	WT
116	115	Ala148Thr	Hmz	2n	WT	WT
	139^a^	WT	Hmz	2n	WT	WT
117	153	WT	Hmz	2n	WT	WT
118	47	WT	Htz	ND	WT	WT
119	12^a^	WT	Htz	ND	WT	WT
120	218	WT	ND	2n	WT	WT
	219	WT	ND	2n	WT	WT
121	220	WT	ND	2n	WT	WT
122	221	WT	ND	2n	WT	WT
123	222	WT	ND	2n	WT	WT
124	223	WT	ND	2n	WT	WT

a Unaffected relatives.

**Table 5:** Mutation detection analysis in p16, p14 and CDK4 genes of pedigrees of melanoma families without neural system tumours (*n*=18)

**Family # (*n*=18)**	**DNA # (*n*=20)**	**P16 sequencing analysis**	**P14 deletion analysis by D9S1748**	**Quantative TaqMan analysis of p14**	**p14 sequencing**	**CDK4 sequencing**
302	104	WT	ND	ND	WT	WT
303	103	WT	ND	ND	WT	WT
305	100	WT	ND	ND	WT	WT
309	112	WT	ND	ND	WT	WT
310	111	Ala148Thr	ND	ND	WT	WT
311	105	WT	ND	ND	WT	WT
312	119	WT	ND	ND	WT	WT
317	116	Ala148Thr	ND	ND	WT	WT
319	114	Ala148Thr	ND	ND	WT	WT
321	15	Ala148Thr	ND	ND	WT	WT
322	124	Ala148Thr	ND	ND	WT	WT
325	78	WT	ND	ND	WT	WT
	79	WT	ND	ND	WT	WT
327	121	WT	ND	ND	WT	WT
	135	WT	ND	ND	WT	WT
329	122	WT	ND	ND	WT	WT
330	140	WT	ND	ND	WT	WT
335	134	Ala148Thr	ND	ND	WT	WT
337	141	WT	ND	ND	WT	WT
338	138	WT	ND	ND	WT	WT

## References

[bib1] Alao JP, Mohammed MQ, Retsas S (2002) The CDKN2A tumour suppressor gene: no mutations detected in patients with melanoma and additional unrelated cancers. Melanoma Res 12: 559–563 12459645 10.1097/00008390-200212000-00005

[bib2] Azizi E, Friedman J, Pavlotsky F, Iscovich J, Bornstein A, Shafir R, Trau H, Brenner H, Nass D (1995) Familial cutaneous malignant melanoma and tumors of the nervous system. A hereditary cancer syndrome. Cancer 76: 1571–1578 8635060 10.1002/1097-0142(19951101)76:9<1571::aid-cncr2820760912>3.0.co;2-6

[bib3] Bahuau M, Vidaud D, Jenkins RB, Bieche I, Kimmel DW, Assouline B, Smith JS, Alderete B, Cayuela JM, Harpey JP, Caille B, Vidaud M (1998) Germ-line deletion involving the INK4 locus in familial proneness to melanoma and nervous system tumors. Cancer Res 58: 2298–2303 9622062

[bib4] Bahuau M, Vidaud D, Kujas M, Palangie A, Assouline B, Chaignaud-Lebreton M, Prieur M, Vidaud M, Harpey JP, Lafourcade J, Caille B (1997) Familial aggregation of malignant melanoma/dysplastic naevi and tumours of the nervous system: an original syndrome of tumour proneness. Ann Genet 40: 78–91 9259954

[bib5] Barrois M, Bieche I, Mazoyer S, Champeme MH, Bressac-de Paillerets B, Lidereau R (2004) Real-time PCR-based gene dosage assay for detecting BRCA1 rearrangements in breast–ovarian cancer families. Clin Genet 65: 131–136 14984472 10.1111/j.0009-9163.2004.00200.x

[bib6] Bertram CG, Gaut RM, Barrett JH, Pinney E, Whitaker L, Turner F, Bataille V, Dos Santos Silva I, Swerdlow AJ, Bishop DT, Newton Bishop JA (2002) An assessment of the CDKN2A variant Ala148Thr as a nevus/melanoma susceptibility allele. J Invest Dermatol 119: 961–965 12406345 10.1046/j.1523-1747.2002.01825.x

[bib7] Bostrom J, Meyer-Puttlitz B, Wolter M, Blaschke B, Weber RG, Lichter P, Ichimura K, Collins VP, Reifenberger G (2001) Alterations of the tumor suppressor genes CDKN2A (p16(INK4a)), p14(ARF), CDKN2B (p15(INK4b)), and CDKN2C (p18(INK4c)) in atypical and anaplastic meningiomas. Am J Pathol 159: 661–669 11485924 10.1016/S0002-9440(10)61737-3 PMC1850553

[bib8] Chin L, Pomerantz J, DePinho RA (1998) The INK4a/ARF tumor suppressor: one gene – two products – two pathways. Trends Biochem Sci 23: 291–296 9757829 10.1016/s0968-0004(98)01236-5

[bib9] Ghimenti C, Fiano V, Chiado-Piat L, Chio A, Cavalla P, Schiffer D (2003) Deregulation of the p14ARF/Mdm2/p53 pathway and G1/S transition in two glioblastoma sets. J Neurooncol 61: 95–102 12622447 10.1023/a:1022127302008

[bib10] Goldstein AM (2004) Familial melanoma, pancreatic cancer and germline CDKN2A mutations. Hum Mutat 23: 630 10.1002/humu.9247 15146471

[bib11] Greene MH (1999) The genetics of hereditary melanoma and nevi. 1998 update. Cancer 86: 2464–2477 10630172 10.1002/(sici)1097-0142(19991201)86:11+<2464::aid-cncr3>3.0.co;2-f

[bib12] Harland M, Meloni R, Gruis N, Pinney E, Brookes S, Spurr NK, Frischauf AM, Bataille V, Peters G, Cuzick J, Selby P, Bishop DT, Bishop JN (1997) Germline mutations of the CDK2 gene in UK melanoma families. Hum Mol Genet 6: 2061–2067 9328469 10.1093/hmg/6.12.2061

[bib13] Hemminki K, Zhang H, Czene K (2003) Familial and attributable risks in cutaneous melanoma: effects of proband and age. J Invest Dermatol 120: 217–223 12542525 10.1046/j.1523-1747.2003.12041.x

[bib14] Hewitt C, Wu CL, Evans G, Howell A, Elles RG, Jordan R, Sloan P, Read AP, Thakker N (2002) Germline mutation of ARF in a melanoma kindred. Hum Mol Genet 11: 1273–1279 12019208 10.1093/hmg/11.11.1273

[bib15] Hussussian CJ, Struewing JP, Goldstein AM, Higgins PA, Ally DS, Sheahan MD, Clark Jr WH, Tucker MA, Dracopoli NC (1994) Germline p16 mutations in familial melanoma. Nat Genet 8: 15–21 7987387 10.1038/ng0994-15

[bib16] Kamb A, Shattuck-Eidens D, Eeles R, Liu Q, Gruis NA, Ding W, Hussey C, Tran T, Miki Y, Weaver-Feldhaus J (1994) Analysis of the p16 gene (CDKN2) as a candidate for the chromosome 9p melanoma susceptibility locus. Nat Genet 8: 22–26 10.1038/ng0994-22 7987388

[bib17] Kaufman DK, Kimmel DW, Parisi JE, Michels VV (1993) A familial syndrome with cutaneous malignant melanoma and cerebral astrocytoma. Neurology 43: 1728–1731 8414022 10.1212/wnl.43.9.1728

[bib18] Laud K, Kannengiesser C, Avril MF, Chompret A, Stoppa-Lyonnet D, Desjardins L, Eychene A, Demenais F, Lenoir GM, Bressac-de Paillerets B, French Herediatary Melanoma Study Group (2003) BRAF as a melanoma susceptibility candidate gene? Cancer Res 63: 3061–3065 12810628

[bib19] Lilischkis R, Sarcevic B, Kennedy C, Warlters A, Sutherland RL (1996) Cancer-associated missense and deletion mutations impair p16^INK4^ CDK inhibitory activity. Int J Cancer 66: 249–254 8603820 10.1002/(SICI)1097-0215(19960410)66:2<249::AID-IJC19>3.0.CO;2-7

[bib20] Loo JC, Paterson AD, Hao A, Shennan M, Peretz H, Sidi Y, Hogg D, Yakobson E (2005) Search for genetic variants associated with cutaneous malignant melanoma in the Ashkenazi Jewish population. J Med Genet 42: e30 15863662 10.1136/jmg.2004.027300 PMC1736056

[bib21] Momand J, Wu HH, Dasgupta G (2000) MDM2 – master regulator of the p53 tumor suppressor protein. Gene 242: 15–29 10721693 10.1016/s0378-1119(99)00487-4

[bib22] Newton Bishop JA, Bataille V, Pinney E, Bishop DT (1994) Family studies in melanoma: identification of the atypical mole syndrome (AMS) phenotype. Melanoma Res 4: 199–206 7950355 10.1097/00008390-199408000-00001

[bib23] Nielsen K, Ingvar C, Masback A, Westerdahl J, Borg A, Sandberg T, Jonsson N, Nagel J, Olsson H (2004) Melanoma and nonmelanoma skin cancer in patients with multiple tumours – evidence for new syndromes in a population-based study. Br J Dermatol 150: 531–536 15030338 10.1111/j.1365-2133.2003.05852.x

[bib24] O'Neill BP, Blondal H, Yang P, Olafsdottir GH, Sigvaldason H, Jenkins RB, Kimmel DW, Scheithauer BW, Rocca WA, Bjornsson J, Tulinius H (2002) Risk of cancer among relatives of patients with glioma. Cancer Epidemiol Biomark Prev 11: 921–924 12223439

[bib25] Paunu N, Pukkala E, Laippala P, Sankila R, Isola J, Miettinen H, Simola KO, Helen P, Helin H, Haapasalo H (2002) Cancer incidence in families with multiple glioma patients. Int J Cancer 97: 819–822 11857361 10.1002/ijc.10107

[bib26] Petronzelli F, Sollima D, Coppola G, Martini-Neri ME, Neri G, Genuardi M (2001) CDKN2A germline splicing mutation affecting both p16(ink4) and p14(arf) RNA processing in a melanoma/neurofibroma kindred. Genes Chromosomes Cancer 31: 398–401 11433531 10.1002/gcc.1159

[bib27] Pomerantz J, Schreiber-Agus N, Liegeois NJ, Silverman A, Alland L, Chin L, Potes J, Chen K, Orlow I, Lee HW, Cordon-Cardo C, DePinho RA (1998) The Ink4a tumor suppressor gene product, p19Arf, interacts with MDM2 and neutralizes MDM2's inhibition of p53. Cell 92: 713–723 9529248 10.1016/s0092-8674(00)81400-2

[bib28] Ranade K, Hussussian CJ, Sikorski RS, Varmus HE, Goldstein AM, Tucker MA, Serrano M, Hannon GJ, Beach D, Dracopoli NC (1995) Mutations associated with familial melanoma impair p16INK4 function. Nat Genet 10: 114–116 7647780 10.1038/ng0595-114

[bib29] Randerson-Moor JA, Harland M, Williams S, Cuthbert-Heavens D, Sheridan E, Aveyard J, Sibley K, Whitaker L, Knowles M, Bishop JN, Bishop DT (2001) A germline deletion of p14(ARF) but not CDKN2A in a melanoma-neural system tumour syndrome family. Hum Mol Genet 10: 55–62 11136714 10.1093/hmg/10.1.55

[bib30] Rizos H, Puig S, Badenas C, Malvehy J, Darmanian AP, Jimenez L, Mila M, Kefford RF (2001) Melanoma-associated germline mutation in exon 1beta inactivates p14ARF. Oncogene 20: 5543–5547 11571653 10.1038/sj.onc.1204728

[bib31] Soufir N, Avril MF, Chompret A, Demenais F, Bombled J, Spatz A, Stoppa-Lyonnet D, Benard J, Bressac-de Paillerets B (1998) Prevalence of p16 and CDK4 germline mutations in 48 melanoma-prone families in France. Hum Mol Genet 7: 209–216 9425228 10.1093/hmg/7.2.209

[bib32] Tachibana I, Smith JS, Sato K, Hosek SM, Kimmel DW, Jenkins RB (2000) Investigation of germline PTEN, p53, p16(INK4A)/p14(ARF), and CDK4 alterations in familial glioma. Am J Med Genet 92: 136–141 10797439 10.1002/(sici)1096-8628(20000515)92:2<136::aid-ajmg11>3.0.co;2-s

[bib33] Ueki K, Ono Y, Henson JW, Efird JT, von Deimling A, Louis DN (1996) CDKN2/p16 or RB alterations occur in the majority of glioblastomas and are inversely correlated. Cancer Res 56: 150–153 8548755

[bib34] Weber JD, Taylor LJ, Roussel MF, Sherr CJ, Bar-Sagi D (1999) Nucleolar Arf sequesters Mdm2 and activates p53. Nat Cell Biol 1: 20–26 10559859 10.1038/8991

[bib35] Xiao ZX, Chen J, Levine AJ, Modjtahedi N, Xing J, Sellers WR, Livingston DM (1995) Interaction between the retinoblastoma protein and the oncoprotein MDM2. Nature 375: 694–698 7791904 10.1038/375694a0

[bib36] Yakobson E, Shemesh P, Azizi E, Winkler E, Lassam N, Hogg D, Brookes S, Peters G, Lotem M, Zlotogorski A, Landau M, Safro M, Shafir R, Friedman E, Peretz H (2000) Two p16 (CDKN2A) germline mutations in 30 Israeli melanoma families. Eur J Hum Genet 8: 590–596 10951521 10.1038/sj.ejhg.5200505

[bib37] Zhu G, Duffy DL, Eldridge A, Grace M, Mayne C, O'Gorman L, Aitken JF, Neale MC, Hayward NK, Green AC, Martin NG (1999) A major quantitative-trait locus for mole density is linked to the familial melanoma gene CDKN2A: a maximum-likelihood combined linkage and association analysis in twins and their sibs. Am J Hum Genet 65: 483–492 10417291 10.1086/302494 PMC1377947

[bib38] Zuo L, Weger J, Yang Q, Goldstein AM, Tucker MA, Walker GJ, Hayward N, Dracopoli NC (1996) Germline mutations in the p16INK4a binding domain of CDK4 in familial melanoma. Nat Genet 12: 97–99 8528263 10.1038/ng0196-97

